# Enhancing the depth and breadth of healthcare services in communities: insights, innovations, and applications

**DOI:** 10.1186/s12913-017-2338-5

**Published:** 2017-07-11

**Authors:** Stephen J. O’Connor

**Affiliations:** Department of Health Services Administration, School of Health Professions, University of Alabama at Birmingham, SHP Building 557, 1720 2nd Avenue South, Birmingham, AL 35294 USA

Regardless of where health reform efforts lead, the major stakeholders of the U.S. health system – patients, providers, payers, employers, communities, and government – will continue to demand greater value. The value concept relates to the quality of benefits and outcomes received for the price that is paid. Essentially, better value emerges with improvements in quality, delivery, access, and patient experience, while reducing or constraining costs.

The drive to advance system performance requires a simultaneous push to innovate in order to make improvements that will result in greater value. Efforts, big and small, to develop value-enhancing innovations and demonstrations are observed in the rapid growth of innovation hubs such as the Center for Medicare and Medicaid Innovation, the VA Center for Innovation, and the Center for Healthcare Innovation. These entities attempt to stay ahead of the changing healthcare environment by working “… to discover, develop, test, and spread new models of care delivery …” [1] that can contribute to more rapid improvements in health system performance and value.

An innovation refers to a good, service, practice, idea [2, 3] or technology [4] used for the first time within an organization, irrespective of whether or not it was used before in another industry or setting. An innovation reflects the newness of the practice to a group of people or an organization in terms of solving problems and improving performance, regardless of when the innovation was first established [5].

Generally, the diffusion of innovations into healthcare organizations from non-health settings arise from: 1) collaborations among healthcare executives and local business leaders, 2) newly hired healthcare executives whose experience is from outside the industry, 3) influential other organizations such as the Institute of Medicine, Joint Commission, Institute for Healthcare Improvement, Agency for Healthcare Research and Quality [6], and the Center for Medicare and Medicaid Services, 4) the importation of best practices and ideas from leading non-healthcare industries, 5) consultants, and 6) innovation centers. However, it is often argued that because healthcare is significantly different from its non-healthcare counterparts, it can be difficult or even futile to try to learn or adopt innovations from them.

Shortell and Kaluzny [7] enumerate the oft-mentioned attributes that serve to make healthcare organizations unique:

- “Defining and measuring output is more difficult.
- The work involved is more highly variable and complex.
- More of the work is of an emergency and nondeferrable nature.
- The work permits little tolerance for ambiguity or error.
- The work activities are highly interdependent, requiring a high degree of coordination among diverse professional groups.
- The work involves an extremely high degree of specialization.
- Organizational participants are highly professionalized, and their primary loyalty belongs to the profession rather than to the organization.
- Little effective organizational or managerial control exists over the group most responsible for generating work and expenditures: physicians.
- Dual lines of authority exist in many health care organizations, particularly hospitals, that create problems of coordination and accountability and confusion of roles” [7], p. 10.

Although they logically refute the uniqueness of each attribute to healthcare, they note that healthcare organizations may be unusual “in that many of them possess all of the characteristics … *in combination*” [7], p. 11. In fact, Wiersinga and Levi suggest, it may “be that the complexity of hospital organizations in which patient care, and often research and education, are fully integrated is too immense or unique to merely duplicate ideas from other industries” [8], p. 425. Nevertheless, the “uniqueness” argument can be detrimental when it encourages healthcare executives to view their work as being so much more difficult or different, that they cannot look to non-healthcare settings for ideas to improve performance.

An openness to new ideas and ways of doing things is essential for applying effective solutions to the hard problems of healthcare today, this requires that healthcare leaders remove the “blinders” to get beyond the barriers of “uniqueness”, broaden their thinking, engage in frank dialogue, and personally encourage a more expansive range of possibilities. This supplement of BMC Health Services Research, by authors James K. Elrod and John L. Fortenberry, Jr., concentrates on innovations that the Willis-Knighton Health System of Shreveport, Louisiana acquired and successfully implemented over time. Although some of these innovative practices are now more commonly observed in the healthcare industry, this was not the case at the time they were adopted and implemented. Indeed, these practices would likely not have been implemented were it not for a deliberate, proactive approach to environmental search and surveillance and an openness to new possibilities derived from beyond the confines of the organization and healthcare industry. The articles show how one health system has been able to get beyond the insular mindset of “uniqueness”.

The first article, “Adaptive reuse in the healthcare industry: repurposing abandoned buildings to serve medical missions,” describes how vacant and unused structures can find new life as healthcare facilities. The article details the process used by the Willis-Knighton Health System to identify, acquire, and renovate abandoned buildings for productive use as functioning structural components within the health system. The benefits of adaptive reuse for both the health system and the community it serves are also described. Although we are witnessing a trend toward greater use of telehealth and e-visits, healthcare is still overwhelmingly facility based – facilities that need to expand in order to support an organization’s growth over time and to house new initiatives embodied in the strategic plan. Adaptive reuse is an approach to fulfilling facility needs that can pay dividends to the healthcare organization and provide a significant asset for stabilizing and revitalizing the community it serves.

The next article, “Centers of excellence in healthcare institutions: what they are and how to assemble them,” provides an overview to the centers of excellence concept and how it was originally conceived as an innovation by the Willis-Knighton Health System many years ago, eventually leading to the development and operation of 11 centers of excellence today. The article provides ideas and guidance for developing centers of excellence.

Centers of excellence are related to the generic strategy of “focus” as presented by Michael Porter [9] and to the notions of service lines, matrix structures, and focused factories. Traditionally, hospitals were exclusively structured as functional designs – organized around the clinical areas and medical specialties (e.g., nursing, pharmacy, medical records, social work, etc.) and nonclinical functional areas (e.g., housekeeping, laundry, food services, engineering, purchasing, billing, etc.). In fact, Malcolm MacEachern’s 1957 book *Hospital Organization and Management* [10] – required reading for hospital administration students of the time – describes only functional designs as appropriate for hospital structures. It makes no mention of designs organized around patients (e.g., woman, children) or their conditions (e.g., cancer, orthopedics, stroke, heart, etc.). The closest MacEachern came to describing a center of excellence or focused service line is in his chapter on specialty institutions where he discussed hospitals for nervous and mental diseases, tuberculosis hospitals, and hospitals for communicable diseases. Thus, because functional designs have been traditionally reinforced in healthcare, the introduction of a centers of excellence innovation was often resisted by clinicians who argued that since excellence was needed to support excellence – what was the point in developing a center of excellence? Although functional designs can be appropriately observed within healthcare organizations today – they are not without limitations. Chief among these is difficulty in effectively coordinating across functions and maintaining a holistic, patient-centered approach.

Regina Herzlinger of Harvard University has written extensively on the focused factory theme in healthcare organizations [11]. This approach is one where “clinical outcomes and patient needs can be improved and money saved, by grouping patients together with similar needs” [12]. Nonetheless, “… there is an absence of detailed case studies that show how the focus concept has been operationalized in hospital settings” [12]. Elrod and Fortenberry clearly address this shortcoming through their center of excellence article.

The third article, “The hub-and-spoke organization design: an avenue for serving patients well” describes another type of design configuration employed within the Willis-Knighton Health System. It provides an overview to the hub-and-spoke structure, how this particular innovation originated within the system, the benefits and risks associated with this type of configuration, and things to consider when planning implementation of such a structure within a healthcare setting.

The final article in the supplement, “Peering beyond the walls of healthcare institutions: a catalyst for innovation,” offers insights into the unique nature of the healthcare industry and how characteristics of the industry itself can contribute to, and perpetuate, inward directed thoughts and mindsets within its executives. This phenomenon can lead to sizeable reductions in the external domains that are surveilled or considered as a source of potential innovative ideas and practices.
